# A Runny Nose Leads to a Rare Diagnosis: Chronic Sinusitis as a Sole Manifestation of Limited Granulomatosis With Polyangiitis

**DOI:** 10.7759/cureus.39203

**Published:** 2023-05-18

**Authors:** Zainab Qudsiya, Zaineb Viqas, Donica L Baker

**Affiliations:** 1 Internal Medicine, St. Luke's Hospital, Chesterfield, USA; 2 Internal Medicine, ECU Health Medical Center, Greenville, USA; 3 Rheumatology, St. Luke's Hospital, Chesterfield, USA

**Keywords:** wegener's granulomatosis, anca-associated vasculitis, chronic sinusitis, chronic granulomatous inflammation, noncaseating granulomas, antineutrophil cytoplasmic antibody (anca) associated vasculitis (aav), recurrent sinusitis, limited gpa, anca, granulomatosis with polyangiitis (gpa)

## Abstract

Granulomatosis with polyangiitis (GPA) is an antineutrophil cytoplasmic antibody (ANCA)-associated necrotizing vasculitis of small to medium-sized blood vessels. The typical presentation is the classic triad of upper airway, pulmonary, and renal involvement. However, it can rarely present with the involvement of a single organ system known as limited granulomatosis with polyangiitis. We present a case of a 53-year-old male with chronic rhinosinusitis as the only manifestation of limited GPA. The diagnosis was established incidentally based on biopsy findings from the paranasal sinuses obtained during functional endoscopic sinus surgery. Subsequent testing revealed a positive cytoplasmic antineutrophilic antibody. No evidence of systemic involvement was noted. Prednisone and azathioprine were initiated leading to significant improvement. Although upper respiratory tract involvement is common in GPA, it is rare for the condition to be limited to this organ system. Our case of limited GPA is distinct in that it represents a rare presentation of this already rare disease.

## Introduction

Granulomatosis with polyangiitis (GPA) is a rare antineutrophil cytoplasmic antibody (ANCA)-associated vasculitis characterized by granulomatous inflammation and pauci-immune necrotizing vasculitis of small- and medium-sized blood vessels. The annual incidence is 11.3 cases/million [1] and is most common among people with European ancestry. Although various organ systems may be involved, the upper respiratory tract, lower respiratory tract, and kidneys are the most commonly and severely affected organs. In 5% of cases, GPA presents as a limited or localized form, wherein the involvement is restricted to a solitary organ system [2]. Compared to severe GPA, limited GPA tends to have a younger mean age of onset (41 years vs. 50 years) and a slight female preponderance (58% vs. 33%) [3]. Here, we present a case of limited GPA in a 53-year-old male with a prolonged history of chronic rhinosinusitis initially misattributed to infectious/allergic etiologies eventually discovered to have a biopsy-proven GPA. This case underscores the importance of considering GPA in the differential diagnosis of chronic recurrent rhinosinusitis unresponsive to symptomatic treatment.

This article was previously presented as a poster abstract at the 2022 Missouri American College of Physicians (ACP) Poster Competition on September 17, 2022.

## Case presentation

A 53-year-old male with chronic rhinosinusitis and hyperlipidemia presented with a documented 14-year duration of recurrent anosmia, purulent nasal discharge, and postnasal drip. This was accompanied by bilateral pressure-like frontal headaches, nonproductive cough, and unintentional weight loss of 8 pounds that occurred over six months prior to presentation. He denied shortness of breath, chest pain, hemoptysis, stridor, hearing loss, skin rash, joint pain, focal weakness, or numbness. Surgical history was notable for nasal polypectomy five years prior to presentation.

On examination, his vitals were a temperature of 36.9°C, heart rate of 78 beats/minute, blood pressure of 120/70 mmHg, respiratory rate of 18 breaths/minute, and oxygen saturation of 98% on room air. Ear, nose, and throat examination revealed crusting of the nasal mucosa, purulent nasal discharge, and a deviated nasal septum. No external nasal deformity was noted. Pulmonary, cardiac, abdominal, neurological, and musculoskeletal exams were unremarkable.

**Figure 1:**
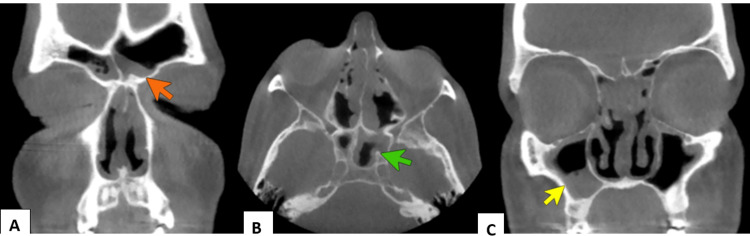
CT of the paranasal sinuses demonstrating chronic inflammation and mucosal thickening of the frontal (orange arrowhead), sphenoid (green arrowhead), and maxillary (yellow arrowhead) sinuses. (A) Coronal view, (B) axial view, and (C) coronal view.

Lab investigations revealed normal complete blood count (white blood cell count = 8.9 thousand/microliter, hemoglobin = 13.8 gram/deciliter, and platelets = 280 thousand/microliter) and metabolic profile (sodium = 141 millimole/liter (mmol/l), potassium = 4 mmol/l, chloride = 103 mmol/l, creatinine = 1.09 milligram/deciliter (mg/dl), and glucose 99 = mg/dl). Inflammatory markers were elevated (erythrocyte sedimentation rate = 119 millimeters/hour and C-reactive protein = 17.1 milligram/liter). QuantiFERON test was done due to travel history to Southeast Asia and was negative. No laboratory evidence of immunodeficiency was demonstrated (immunoglobulins A, G, and M and pneumococcal antibodies were in the normal range). Computed tomography (CT) scan of the paranasal sinuses revealed chronic pansinusitis and septal edema (Figure 1).

**Figure 2:**
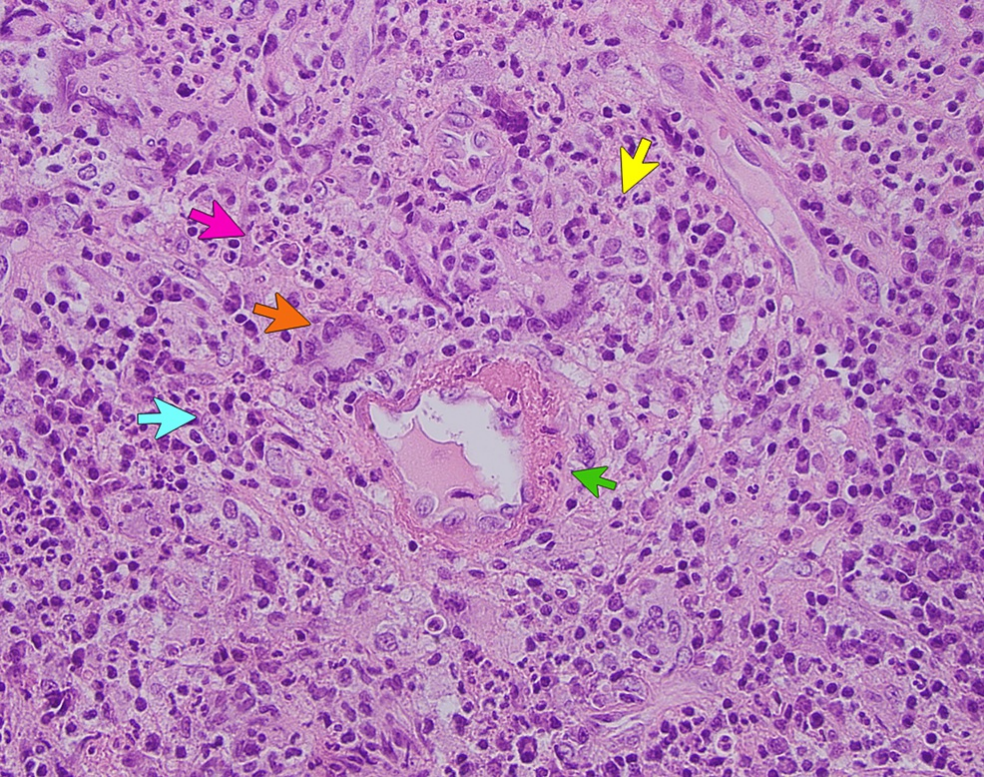
Histopathological exam of biopsy from the paranasal sinus (hematoxylin and eosin stain) demonstrating granulomatous inflammation with necrosis (pink arrow), inflammatory cells-neutrophils (yellow arrow), plasma cells (blue arrow), and multinucleated giant cells (orange arrow). Vasculitis with fibrinoid changes of the vessel wall (green arrow).

**Figure 3:**
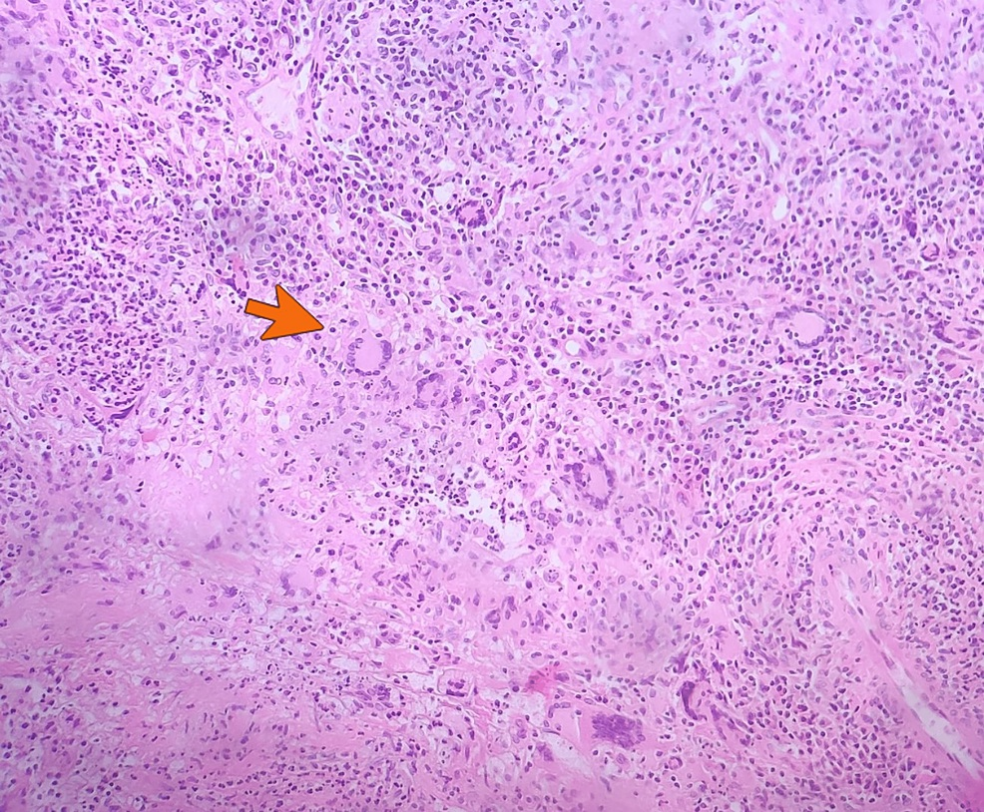
Histopathological exam of nasal specimen biopsy (hematoxylin and eosin stain) demonstrating granulomatous inflammation with multinucleated giant cells (orange arrow).

**Figure 4:**
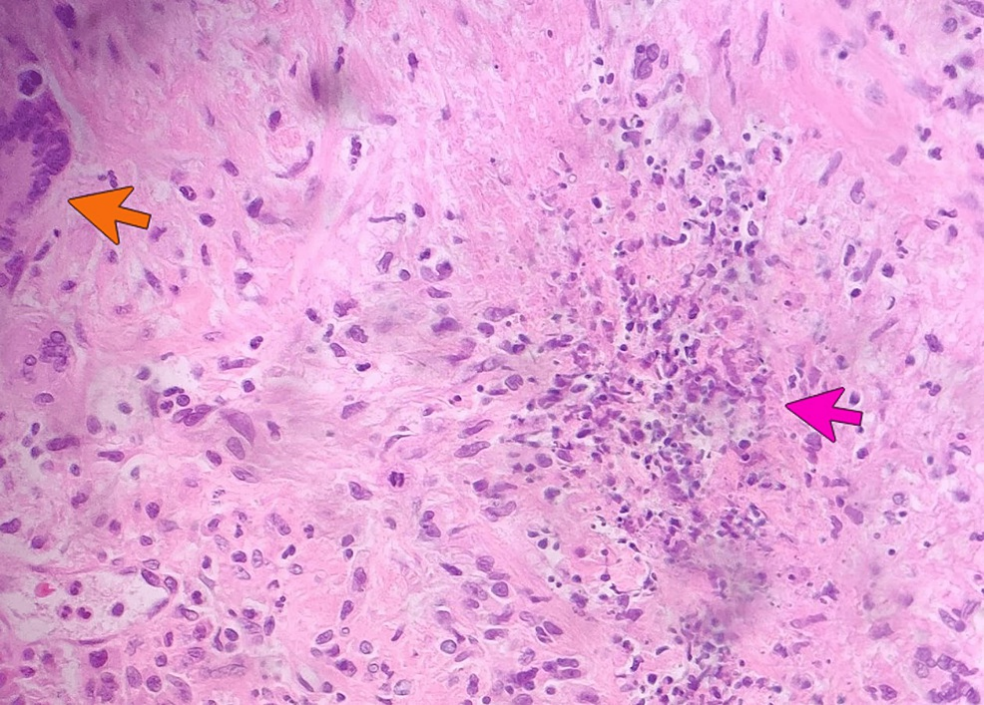
Histopathological exam of nasal biopsy (hematoxylin and eosin stain) demonstrating necrotizing granuloma (pink arrow) with surrounding multinucleated giant cells (orange arrow).

Upper respiratory symptoms recurred despite symptomatic management with decongestants, intranasal steroids, anti-leukotriene agents, multiple courses of antibiotics, and immunotherapy (interleukin-4/interleukin-13 monoclonal antibody) prescribed by his otolaryngologist and allergist. A month before the presentation, he underwent functional endoscopic sinus surgery. Biopsy from sinus and nasal contents incidentally revealed acute on chronic inflammation, non-caseating granulomas, necrosis, multinucleated giant cells, and vasculitis (Figures 2-4). Periodic acid-Schiff (PAS) and acid-fast bacilli (AFB) stains were negative for fungi and mycobacteria, respectively.

Further testing revealed positive antineutrophilic cytoplasmic antibodies (C-ANCA), 1:40 titer, positive myeloperoxidase (MPO), and negative proteinase 3 (PR3). No evidence of systemic involvement was noted. CT of the chest was reported normal with no evidence of cavitation, nodules, alveolar opacities, or pleural effusion. No evidence of renal dysfunction or proteinuria was noted (urine microalbumin to creatinine ratio was in the normal range). Based on C-ANCA positivity, lack of systemic involvement, and biopsy findings, he was subsequently diagnosed with limited GPA of the upper respiratory tract. Therapy with oral prednisone (initial dose of 15 milligrams (mg), tapered by 1 mg per month) and azathioprine (initial dose of 50 mg, gradually up-titrated to 150 mg) was initiated with significant improvement in clinical manifestations.

## Discussion

The upper respiratory tract is the most commonly affected organ in limited GPA, although cases confined to the lungs, kidneys, and eyes have also been noted. Limited GPA of the upper respiratory tract presents with recurrent rhinosinusitis, purulent nasal discharge, crusting, nasal ulcers, otitis media, mastoiditis, and hearing loss. These manifestations of limited GPA can easily be misdiagnosed as allergic and infectious sinusitis, which can lead to a delay in the recognition of this condition, as was the case with our patient. Such delay can be detrimental, as, despite a lack of systemic involvement, limited GPA is associated with a higher risk of local tissue destruction. Such damage can manifest in the form of a saddle nose deformity, septal perforation, and/or orbital wall destruction [3,4]. Fortunately, our patient did not demonstrate any evidence of local destructive lesions clinically or on CT of the paranasal sinuses.

The diagnosis of GPA depends on serology, clinical presentation, and histopathology [5]. C-ANCA is highly specific for GPA (99%), correlates with disease activity, and is commonly associated with PR3 antibodies. In 9% of cases, patients are MPO-ANCA positive, which is associated with limited expression, a lower risk of severe organ involvement, and require less aggressive immunotherapy [6-9]. Despite having a prolonged course, our patient, who was also MPO-ANCA positive, did not exhibit progression to systemic involvement. It is worth noting, however, that only 46% of cases of limited GPA are ANCA-positive. In such cases, the histopathological examination of the biopsy from the affected organ is crucial to establishing a diagnosis. The classic findings on histopathology include vasculitis of small and medium-sized blood vessels, granulomatous inflammation consisting of leukocytes, lymphocytes, plasma cells, dendritic cells, eosinophils, and multinucleated giant cells, and necrosis. In general, upper respiratory tract biopsies taken are nonspecific and carry low diagnostic yield, with typical findings observed in only 25-50% of cases [10]. However, our patient’s nasal mucosa and paranasal sinus biopsy specimens demonstrated all the typical findings of GPA sufficient to confirm the diagnosis, which is unusual.

Management of GPA depends on the presence of life or organ-threatening disease, defined as alveolar hemorrhage, glomerulonephritis, central nervous system vasculitis, mononeuritis multiplex, cardiac involvement, mesenteric, limb, or digit ischemia. Our patient classifies as a non-severe GPA as he lacked an organ or life-threatening disease. In such non-severe cases, the American College of Rheumatology (ACR) favors the use of less toxic therapies first. Recommended induction therapy is glucocorticoids with methotrexate. Other options include a combination of glucocorticoids with azathioprine, rituximab, cyclophosphamide, or mycophenolate, followed by maintenance therapy [11]. Patients with limited GPA have a notable tendency for relapse, impacting 46% of individuals. Complete remission is observed in 34% of patients. The risk of disease progression is relatively low, with only 10% of patients progressing to generalized disease within a median duration of six years. However, despite a low risk of systemic involvement, localized organ damage is frequently observed, primarily due to bone destruction or space obstruction, affecting 66% of cases. These findings underscore the importance of early diagnosis in managing limited GPA [4].

## Conclusions

This case illustrates the diagnostic challenge of recognizing limited GPA. Isolated upper respiratory tract involvement is mimicked by infectious/allergic etiologies often leading to a delay in diagnosis, such as in our case. This entity should be considered in chronic sinonasal disease refractory to symptomatic management and prompt evaluation with ANCA and histopathology to guide diagnosis. Due to its association with a higher risk of local tissue destruction, early recognition and treatment of Limited GPA can prevent complications.
